# Influence of *Salvia miltiorrhizae* on the Mesenteric Lymph Node of Rats with Severe Acute Pancreatitis or Obstructive Jaundice

**DOI:** 10.1155/2009/675195

**Published:** 2010-02-15

**Authors:** Zhang Xiping, Zhang Jie, Ye Shuyun, Wang Qili, Feng Guanghua, Pan Yan

**Affiliations:** ^1^Department of General Surgery, Hangzhou First People's Hospital, Zhejiang Province, Hangzhou 310006, China; ^2^The First College of Clinical Medicine, Zhejiang Traditional Chinese Medicine University, Zhejiang Province, Hangzhou 310053, China

## Abstract

*Objective*. To observe the effect of *salvia miltiorrhizae* injection on inflammatory mediator levels and mesenteric lymph nodes in severe acute pancreatitis (SAP) and obstructive jaundice (OJ) rats and explore the protective mechanism of *salvia miltiorrhizae* on the lymph nodes of these rats. *Methods*. A total of 288 rats were used in SAP-associated and OJ-associated experiments. The rats were randomly divided into sham-operated group, model control group, and treated group. At various time points after operation, the pathological changes in mesenteric lymph nodes of rats in each group were observed, respectively. *Results*. The pathological severity scores in lymph nodes of SAP rats in treated group were significantly lower than those in model control group (*P* < .05) while the pathological changes in lymph nodes of OJ rats in treated group also showed varying degrees of mitigation. *Conclusion*. *Salvia miltiorrhizae* can exert protective effects on the lymph nodes of SAP or OJ rats via a mechanism that is associated with reducing the contents of inflammatory mediators in blood.

## 1. Introduction

Severe acute pancreatitis (SAP) and obstructive jaundice (OJ), characterized by multiple complications and high mortality rate, have become one of the important medical problems that need to be overcome over the years. The pathogenesis of two different diseases is very complicated. In recent years, it has been found that the main contributing factors to the occurrence of multiple organ dysfunction in SAP and OJ include the massive release of various inflammatory mediators, the dysregulation of the equilibrium between proinflammatory and antiinflammatory effects, and the disturbance of immune function [1–3].

Therefore, it is important to inhibit the excessive release of inflammatory mediators [4, 5] and restore the function of the immune system [6] in the therapy of SAP or OJ. Some clinical studies have shown that *salvia miltiorrhizae*, as an auxiliary drug, has some therapeutic effects on SAP and OJ. *Salvia miltiorrhizae* is a common Chinese herbal medicine [7, 8]; it is able to improve the clinical symptoms in patients with SAP and significantly shorten the recovery duration of urinary amylase and liver function damage, the remission duration of abdominal pain, as well as the duration of hospitalization [9, 10].

Moreover, *salvia miltiorrhizae* can also protect intestinal mucosa, reduce the translocation of bacteria and endotoxins, enhance the immunity, improve operative safety, and shorten the recovery duration in OJ patients [11]. In our previous reports, we have detected the serum contents of some inflammatory mediators in SAP and OJ rats (including endotoxin, PLA_2_, and TNF-*α*) [10, 12–14] and found them significantly increased in model control groups of rats. In this study, we observed the pathological changes in the mesenteric lymph nodes of SAP and OJ rats, explored the protective effect and mechanism of *salvia miltiorrhizae* injection on lymph nodes, and further clarified the therapeutic significance of inflammatory mediators in SAP and OJ. This study was closely related to our serial previous reports.

## 2. Materials and Methods

### 2.1. Materials

288 healthy male SD rats of clean grade, weighing between 270 and 330 g, were provided by the Laboratory Animal Research Center of the Zhejiang University of Traditional Chinese Medicine (Hangzhou, China); sodium taurocholate and sodium pentobarbital were purchased from Sigma Corporation Ltd. (St. Louis, MO, USA); *salvia miltiorrhizae* injection (each 10 mL vial contains active components equivalent to 15 g of the original medicine) was purchased from Chiatai Qingchunbao Pharmaceutical Co., Ltd. (Hangzhou, China).

### 2.2. Methods 

#### 2.2.1. Animal Grouping

108 rats were used for SAP-associated experiments and randomly divided into sham-operated, model controlgroup, and treated group (*n* = 36), which were further randomly subdivided into 3-hour, 6-hour, and 12-hour groups (*n* = 12) according to time points after operation; another 180 rats were utilized for OJ-associated experiments and randomly divided into sham-operated group, model control group, and treated group (*n* = 60), which were further randomly subdivided into 7-day, 14-day, 21-day, and 28-day groups (*n* = 15) according to time duration after operation.

#### 2.2.2. Preparation of SAP Models and Associated Therapeutic Regimen

The rats were anesthetized with an intraperitoneal injection of 2.5% sodium pentobarbital (0.2 ml/100 g). Under aseptic conditions, the thigh skin was cut open to expose femoral vein and a transfusion passage was established, through which continuous infusion was maintained using a microinfusion pump (1 ml/h/100 g). Subsequently, a median abdominal wall incision was made to expose duodenal papilla, and a No. 5 syringe needle was used to prick a small hole in the mesenteric avascular area. The epidural catheter was first inserted into duodenal cavity via the hole and then placed into the bile-pancreatic duct towards the direction of papilla. The catheter head was temporarily clamped using a microvascular clamp, and another microvascular clamp was used to occlude the common bile duct at the confluence of hepatic ducts to prevent a backflow of injected drugs into the liver. After connecting the epidural catheter end with the transfusion converter, 3.5% sodium taurocholate (0.1 ml/100 g) was transfused at a flow rate of 0.2 ml/min using a microinjection pump (produced by Zhejiang University, Hangzhou, China). After completing the transfusion, microvascular forceps and epidural catheter were maintained for further 4 minutes and then removed. A checkup was then conducted to see whether bile leakage was present. After suturing the hole in the lateral wall of the duodenum, the abdominal cavity was closed conventionally. Sham-operated group was performed just by moving the pancreas and duodenum after opening the abdominal cavity. Fifteen minutes after successful operation, a single dose of *salvia miltiorrhizae* injection (0.4 ml/100 g body weight) was given via femoral vein to rats in the treated group while equal volume of physiological saline solution was used in the sham-operated and the model control groups [10–16]. Continuous infusion of physiological saline solution using a microinjection pump was then maintained until the end of the 3-hour, 6-hour, and 12-hour observation periods in the corresponding groups.

#### 2.2.3. Preparation of OJ Models and Associated Therapeutic Regimen

After rats were anesthetized with an intraperitoneal injection of 2.5% sodium pentobarbital (0.2 ml/100 g), the abdominal cavity was opened to identify and dissociate common bile duct along the hepatoduodenal ligament. For rats in the model control group and the treated group, the proximal end of common bile duct was double-ligated with surgical threads, common bile duct was cut off, and a layered suture of the abdominal wall was performed to close the abdominal cavity. For rats in the sham-operated groups, common bile duct was only dissociated but not ligated, and a layered suture of the abdominal wall was also performed to close the abdominal cavity. An intraperitoneal injection of *salvia miltiorrhizae* injection at a dose of 0.2 ml/100 g/d was given to rats in the treated group while equal volume of physiological saline solution was used in the sham-operated and the model control groups [10–16]. Injection was maintained until the end of the 7-day, 14-day, 21-day, and 28-day observation periods in the corresponding groups.

#### 2.2.4. Specimens Collection

At the corresponding time points after operation, SAP or OJ rats were anesthetized with 2.5% sodium pentobarbital and mercifully killed. The tissue specimens of mesenteric lymph node were collected and then observed the pathological changes as well as pathological severity scores, respectively. At 12 hours after operation in SAP experiment, two rats were randomly selected from each group, and the ultrastructural changes in the mesenteric lymph node were observed under an electron microscope.

#### 2.2.5. Statistical Analysis

The compiled data were first input into the Excel sheet and then read into SPSS15.0 for further analysis. Normal data were expressed as means (standard deviation) while nonnormal data were expressed as medians (interquartile range). Analysis of variance and pairwise comparisons were used for normal data, whereas nonnormal data were subjected to nonparametric test, among which Kruskal-Wallis *H* test was used for pairwise comparisons and Mann-Whitney *U* test for multiple comparisons. Yates' chi-square test (*χ* ^2^) was used for intergroup comparisons of mortality rates.

## 3. Results

### 3.1. SAP-Associated Experiments 

#### 3.1.1. Pathological Changes in Lymph Nodes

**(1) Sham-Operated Group**

Grossly, the morphology of lymph nodes was normal. Under light microscopy, the morphology and structure of lymph nodes were roughly normal. The expansion of lymph sinuses, the hyperplasia of sinus cells as well as the filtration of few lymphocytes, and eosinophilic granulocytes in the sinuses were observed in some rats. Under electron microscopy, the morphology and structure of lymph nodes were roughly normal.

**(2) Model Control Group**

**Figure 1:**
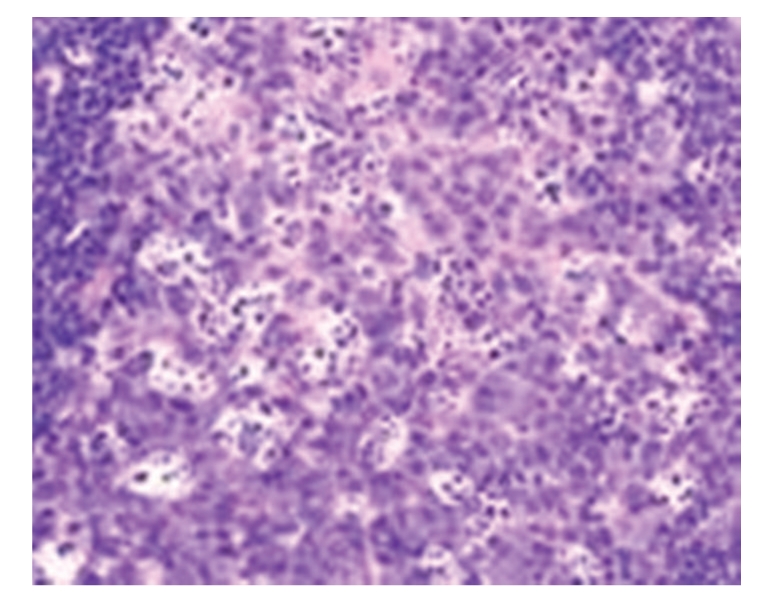
Model control group: 12 hours in SAP rat focal necrosis of germinal center of lymph node, HE × 400.

**Figure 2:**
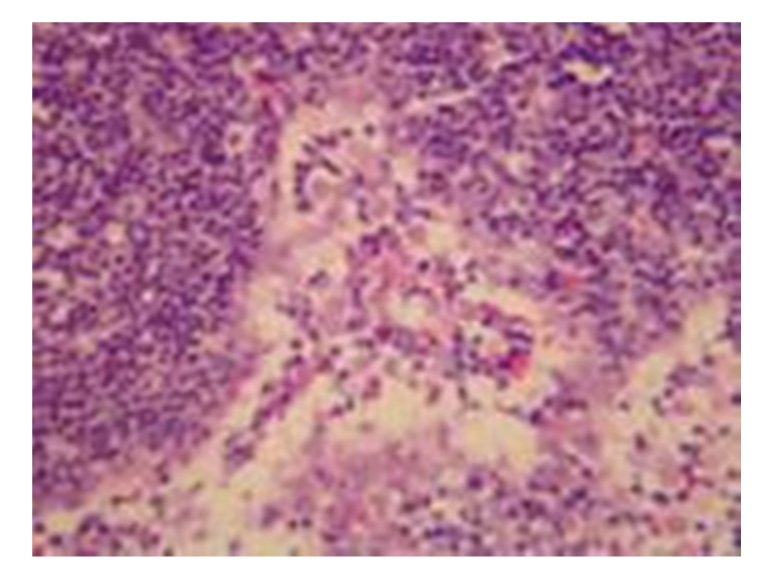
Model control group: 12 hours in SAP rat expansion of lymph sinuses, hyperplasia of sinus cells, and obvious infiltration of many inflammatory cells, HE × 200.

Grossly, the morphology of lymph nodes was roughly normal. Under light microscopy, slight enlargement and congestion of interstitial capillaries were observed. Under electron microscopy, mitochondrial swelling, disappearance and vacuolation, as well as lymphocyte necrosis and apoptosis were seen; see Figures 1 and 2.

**(3) Treated Group**

**Figure 3:**
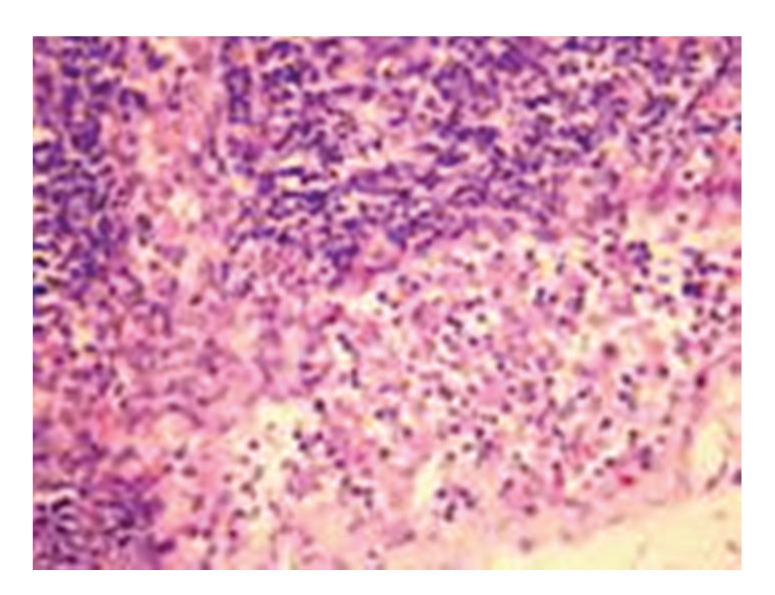
Treated group: 12 hours in SAP rat expansion of lymph sinuses, hyperplasia of sinus cells, and infiltration of small part of some lymphocytes, HE × 200.

Grossly, the morphology of lymph nodes was roughly normal. Under light microscopy, the pathological changes were slightly mitigated when compared with those in model control group. Under electron microscopy, the swelling, disappearance, and vacuolation of few mitochondria were seen; see Figure 3.

#### 3.1.2. Comparison of the Pathological Severity Scores in Lymph Nodes

The pathological score of lymph nodes was conducted according to the standard reported in the literature [17]. At all time points after operation, no significant difference in the pathological severity scores in lymph nodes was noted between sham-operated group and model control group (*P*> .05); at 6 hours after operation, the pathological score in treated group was significantly higher than that in sham-operated group (*P*< .05); at 3 and 6 hours after operation, the pathological severity scores in treated group were significantly lower than those in model control group (*P*> .05); see Table 1.

### 3.2. OJ-Associated Experiments 

#### 3.2.1. Pathological Changes in Lymph Nodes

**(1) Sham-Operated Group**

Grossly, the morphology of lymph nodes was normal. Under light microscopy, no marked difference in pathological changes was observed among each time points after operation; the morphology and structure of lymph nodes were roughly normal; the enlargement of the follicular germinal centers and the hyperplasia of sinus cells were seen in few rats.

**(2) Model Control Group**

**Figure 4:**
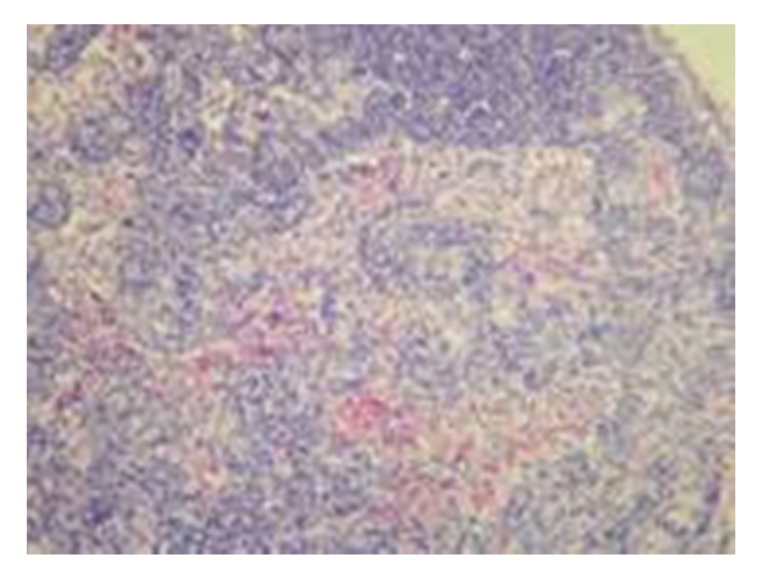
Model control group: 28 days in OJ rat hyperplasia of sinus cells, infiltration of a little eosinophile granulocytes, and a little hemorrhage, HE × 200.

Grossly, lymph nodes became yellow in half of the rats on 7 days after operation and in the majority of rats on 14, 21, and 28 days after operation; the texture of lymph nodes showed no alterations at all time points after operation. Under light microscopy, no marked difference in pathological changes was observed among each time points after operation; on 7 days after operation, the enlargement of the follicular germinal centers and the hyperplasia of sinus cells were seen in the majority of rats, and few rats showed no obvious pathological changes in lymph nodes; on 14 days after operation, the enlargement of the follicular germinal centers and the hyperplasia of sinus cells were seen; on 21 and 28 days after operation, the enlargement of the follicular germinal centers and the hyperplasia of sinus cells were seen in the majority of rats, and spotty necrosis could be seen in the mantle zone and germinal centers; see Figure 4.

**(3) Treated Group**

**Figure 5:**
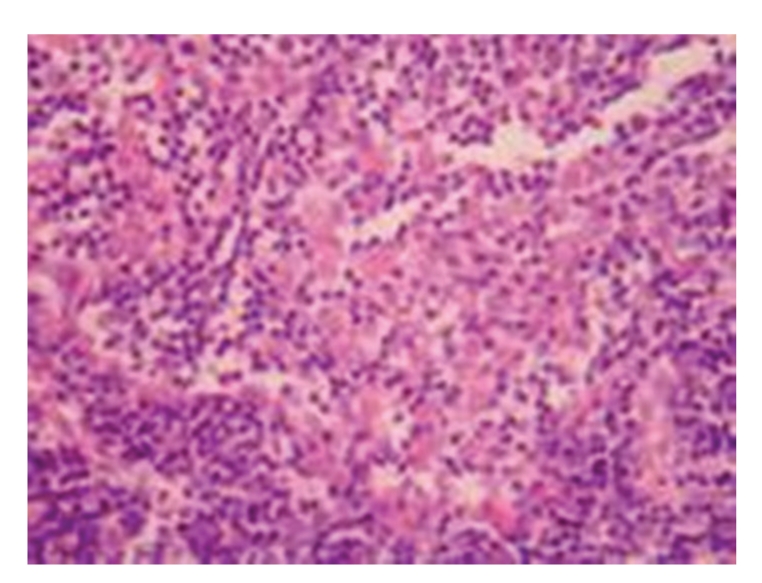
Treated group: 28 days in OJ rat expansion of lymph sinuses, hyperplasia of sinus cells, and infiltration of inflammatory cells, HE × 200.

Grossly, the pathological changes showed no marked difference on 7 days after operation when compared with those in model control group; on 14 days after operation, lymph nodes became yellow but showed no marked difference compared with those in model control group; on 21 and 28 days after operation, the pathological changes showed no marked difference compared with those in model control group. Under light microscopy, no marked difference in pathological changes was observed among each time points after operation; at all time points after operation, the boundary of the follicular germinal centers in lymph nodes was clear; the enlargement of the follicular germinal centers and the hyperplasia of sinus cells were seen in the majority of rats; and few rats showed no obvious pathological changes in lymph nodes; see Figure 5.

#### 3.2.2. Comparison of the Pathological Severity Scores in Lymph Nodes

The pathological score of lymph nodes was conducted according to the standard reported in the literature [17]. On 14, 21, and 28 days after operation, the pathological severity scores in lymph nodes in model control group were significantly higher than those in sham-operated group (*P*< .05); at all time points after operation, the pathological severity scores in treated group were significantly higher than those in sham-operated group (*P*< .05), and no significant difference was noted between treated group and model control group (*P*< .05); see Table 2.

## 4. Discussion

Although the pathogenesis of SAP and OJ is very complicated, their ultimate outcomes are always local and systemic inflammatory response [18] that will eventually induce MODS (multiple organ dysfunction syndrome) and MOF (multiple organ failure). Besides being involved in the infections and inflammatory damage secondary to SAP or OJ, the translocation of intestinal bacteria is also closely related with the decline in the defense function of mesenteric lymph nodes [19–22]. Therefore, it is of important significance to protect mesenteric lymph nodes in the therapy of SAP and OJ. *Salvia miltiorrhizae* has advantages of lower cost, extensive pharmacological effects, and fewer side effects, and it is able to prevent calcium overload, scavenge oxygen free radicals, protect against inflammation, and improve microcirculation. Some animal experimental results have shown [23–25] that *salvia miltiorrhizae* is able to significantly reduce the excessive levels of bilirubin and endotoxin, antagonize SAP- or OJ-induced increase in the intestinal permeability and intestinal bacterial translocation, effectively inhibit the production of inflammatory mediators, and thereby protect multiple organs. The results of our previous study showed that the survival rates of SAP and OJ rats in treated group were higher than those in model control group [10–16], suggesting that *salvia miltiorrhizae* has some therapeutic effects on SAP or OJ rats. However, no statistical difference in the survival rates of rats was noted between treated group and model control group. We surmise that this may be because the sample size is too small.

In this study, we found that, on 14, 21, and 28 days after operation, the pathological severity scores in the lymph nodes of OJ rats in model control group were significantly higher than those in sham-operated group (*P*< .05), indicating that OJ can induce pathological injury in the lymph nodes of rats. Under electron microscopy, the necrosis and apoptosis of lymphocytes as well as the swelling of mitochondrial cristae were observed in the lymph nodes of SAP rats in model control group, suggesting that SAP can also induce pathological injury in the lymph nodes of rats. Since both SAP and OJ can induce lymph node injury, they may threaten the stability of the body's immune function. Thus, it is of important significance to protect lymph nodes with drugs.

Endotoxin, as one of the most important inflammatory mediators that are involved in the pathogenesis of SAP and OJ, may play important roles in the initiation of SIRS/MOD and the aggravation of AP [26]. It can directly stimulate the Kupffer cells to release inflammatory mediators, including oxygen free radicals, TNF-*α*, IL-6, and IL-8, and thereby aggravate the body's inflammatory response [27, 28]. TNF-*α* is the most important factor that mediates the toxic effects of endotoxin. The excessive release of TNF-*α* may induce a variety of pathological injuries. PLA_2_ is a Ca^2+^-dependent enzyme [29]. In SAP and OJ, PLA_2_ is activated and massively released into the blood. After reaching various organs via the blood circulation, PLA_2_ can destroy the lipid membrane of cells and cause direct cytotoxicity. Some studies showed that, in SAP complicated with MODS, PLA_2_ could induce severe damage and dysfunction of lung, liver, kidney and heart [30] while its antagonists could improve the pathological changes in SAP [31]. The results of our previous study showed that the contents of inflammatory mediators such as endotoxin, TNF-*α* and PLA_2_ in model control group were significantly higher than those in sham-operated group [10, 12–14], suggesting that inflammatory mediators were massively released in SAP and OJ. Those results indicated that *salvia miltiorrhizae* was able to significantly reduce the levels of endotoxin, TNF-*α* and PLA_2_ in SAP and OJ rats. This may be because *salvia miltiorrhizae* can effectively inhibit the release of endotoxin and thereby reduce the production of other inflammatory mediators such as TNF-*α*, IL-6, and IL-8. Concomitant with the decline in the levels of inflammatory mediators, at 3 and 6 hours after operation, the pathological scores in the lymph nodes of SAP rats in treated group were significantly lower than those in model control group (*P*< .05). Under light microscopy, we found that the boundary of the follicular germinal centers in lymph nodes of OJ rats in treated group was clear, and few lymph nodes revealed no abnormality. Compared to model control group, the pathological changes in treated group were improved. Therefore, we believe that inflammatory mediator levels are correlated with the severity of pathological changes in lymph nodes in SAP and OJ rats. In other words, inflammatory mediators can induce pathological damage in the lymph nodes of rats, whereas *salvia miltiorrhizae* can exert protective effects on the lymph nodes of rats through reducing the levels of inflammatory mediators. Some studies have proved that *salvia miltiorrhizae* can antagonize endotoxin-induced effects [32]. We think that *salvia miltiorrhizae* exerts its protective effect on lymph nodes mainly through the following two aspects.

**Antiinflammatory Effect**

The inflammatory mediators produced in SAP and OJ can cause intestinal mucosal damage. Both endotoxin and TNF-*α* can directly induce local ischemia and increased permeability of intestinal mucosa, mediate the massive production of cytokines and pro-inflammatory response, and thereby induce the necrosis and apoptosis of cells as well as multiple organ dysfunction [33–37]. Through inhibiting the production of endotoxin and blocking the pathways through which inflammatory cytokines are produced, *salvia miltiorrhizae* is able to effectively modulate the gut microenvironment, protect mesenteric lymph nodes, and restore intestinal barrier function. Our series of studies, which have been or will be reported in other papers, have proved that *salvia miltiorrhizae* is able to protect intestinal mucosa, providing a further basis for this hypothesis.

**Improving the Body's Immune Function**

It has been pointed out in some studies [38] that endotoxin can affect the body's immune function through inducing the production of inflammatory mediators. The decline in immune function can impair the body's antiinfection ability, increase the translocation of intestinal bacteria and endotoxins, and aggravate inflammatory response and tissue damage. Therefore, suppressing the production of inflammatory mediators, such as endotoxin, TNF-*α*, and PLA_2_, can block inflammatory mediator-induced damage to the immune defense system, which is important for stabilizing the body's immune system. Since *salvia miltiorrhizae* perhaps has this effect, it is worthy to deeply study its effect on the immune function in the future.

In short, *salvia miltiorrhizae* is able to protect the mesenteric lymph nodes of SAP and OJ rats through reducing the levels of inflammatory mediators in blood. This effect plays a positive role in stabilizing the body's immune function, protecting intestinal mucosa barrier, and reducing the translocation of bacteria. As the pharmacological effects of *salvia miltiorrhizae* are further clarified, *salvia miltiorrhizae* will be more widely used to treat SAP and OJ.

**Note**

We claimed that this paper was original and would not have any financial interest in a company or its competitor and that all authors meet standard for authorship. We abided by the ethics in this animal experiment study. The ethics committee approval of our hospital was secured for the animal study reported, and all rats have not been abused and executive merciful killing when the observing time in this study was over was conducted.

## Figures and Tables

**Table 1:** Comparison of pathological score of lymph node in SAP groups (*M*(*Q*_*R*_)).

Indexes	Sham-operated group			Model control group			Treated group		
	3 hours	6 hours	12 hours	3 hours	6 hours	12 hours	3 hours	6 hours	12 hours
Pathological score	2.00	2.00	2.00	2.00	2.00	2.00	1.00^+^	1.00***^+^	1.00
	(1.00)	(0.00)	(0.50)	(1.00)	(0.00)	(1.00)	(2.00)	(1.50)	(1.00)

Note. Compare to sham-operated group, ***P< .001; compare to model control group, ^+^ *P*< .05.

**Table 2:** Comparison of pathological score of lymph node in OJ groups (*M*(*Q*_*R*_)).

Indexes	Sham-operated group				Model control group				Treated group			
	7 days	14 days	21 days	28 days	7 days	14 days	21 days	28 days	7 days	14 days	21 days	28 days
Pathological score	0.00	0.00	0.00	0.00	1.00	1.00*	1.00*	1.50*	1.00*	2.00*	1.00*	1.00*
	(2.00)	(1.00)	(1.00)	(1.00)	(2.00)	(1.00)	(2.00)	(1.00)	(1.00)	(1.50)	(0.00)	(1.00)

Note. Compare to sham-operated group, *P< .05.
